# Prof. Peter Erich Dieter, PhD: 22.02.1952 – 25.12.2019

**DOI:** 10.3205/zma001295

**Published:** 2020-02-17

**Authors:** Eckhart G. Hahn

**Affiliations:** 1 Gesellschaft für Medizinische Ausbildung (GMA), Erlangen, Germany; 2 GMS Journal for Medical Education (GMS JME), Erlangen, Germany

## Obituary

**Figure 1:**
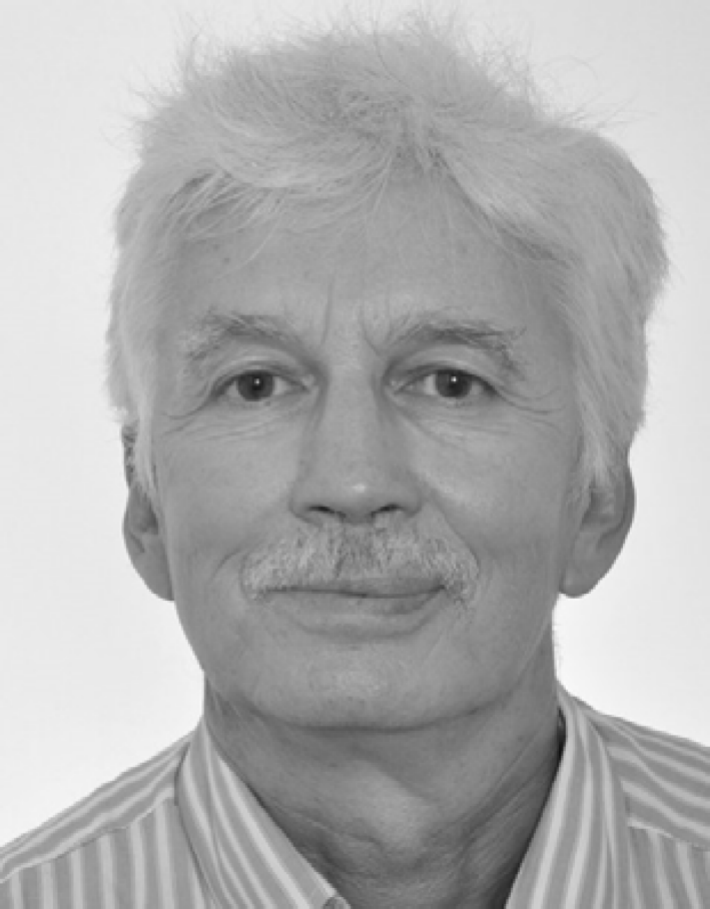
Prof. Peter Erich Dieter, PhD: 22.02.1952 – 25.12.2019

Our colleague Prof. Peter Erich Dieter (see figure 1 (Fig. 1)), PhD passed away in his 68^th^ year on Christmas Day. He had been diagnosed with a neuroendocrine tumor (NET) in July of 2017. He is survived by his wife Kathryn, his daughter Claudia, and his brothers Rolf and Karlheinz.

We in the Association for Medical Education (Gesellschaft für Medizinische Ausbildung, GMA) mourn a dear friend and colleague, who had dedicated himself to the appreciation, advancement and quality of medical education. At Carl Gustav Carus Medical School of Dresden Technical University he had, since 1999, developed, implemented, certified and evaluated a reform curriculum DIPOL^®^ in cooperation with Harvard International, and also made himself a name in Germany, Europe and outside Europe. He was a member of the GMA from 2000-2017, a member of GMA’s board of directors, and vice-president from 2003-2011. During this time the GMA underwent fundamental changes, and Peter, with his pedagogic competence and his profile as a teacher and investigator in molecular biochemistry, made fundamental contributions to the board of directors’ program. Likewise, in 2005, he contributed to the new edition of the “GMS Zeitschrift für Medizinische Ausbildung” (now GMS Journal of Medical Education) as an open access journal and was an active editorial member and reviewer up to 2019. With his close connections to the German Association of Medical Faculties (“Deutscher Medizinischer Fakultätentag, MFT”) he played an important role as intermediary for and contributor to the National Competence-Based Learning Catalogue for Medicine (Nationaler Kompetenzbasierter Lernzielkatalog Medizin, NKLM) and for Dentistry (NKLZ) in Germany, jointly developed by the GMA and the MFT. Notwithstanding it was characteristic for him that he never wanted to outshine his colleagues and preferred to remain in the background. The board of directors of the GMA, during this period of dynamic change until 2011, could always rely on his support and loyalty.

Peter Erich Dieter was born in 1952 in Neustadt an der Weinstraße (Rheinland-Pfalz) where he also graduated from high school (Abitur) in 1970. His mother thought he would make a good teacher, and following her advice he enrolled at the Landau University of Education in pursuit of a teaching degree for secondary schools. In 1972 he decided to change track and moved to the Albert-Ludwig-University at Freiburg im Breisgau where he gained a degree in biology (Bachelor of Science) in 1978 with a diploma thesis on “Active uptake of Ca^2+^ in mitochondria and microsomes of corn”. He continued teaching biology, chemistry and mathematics at a private secondary school in Freiburg and worked on his PhD degree (Dr. rer. nat., biology) at Freiburg University which he received in 1981 (Ca^2+^/calmodulin-dependent enzymes in higher plants). In 1981 he was employed as a postdoctoral fellow at the University of Freiburg and was a guest investigator at Canberra University, Canberra, Australia from 1986-1987, and at the National Institutes of Mental Health, Bethesda, Maryland, in 1989. In 1991 he received his “Habilitation” in medical biochemistry for his research on the “Regulation of the synthesis of eicosanoids and superoxide in macrophages of the liver” and in 1992 was offered a position as a university lecturer in medical biochemistry.

On December 27, 1996 he married Kathryn Asman, a mezzo-soprano opera singer from the USA, who henceforth would accompany him in his professional career and was often helpful in his dealings with international institutions. He delighted in moderating classical concerts with his wife and her pianists all over Germany.

In 1997 he was appointed Professor of Biochemistry/Molecular Biology at the Institute of Physiological Biochemistry, Carl Gustav Carus Medical School, Technical University Dresden, Germany. Soon thereafter he was appointed program director for the project “Reform of the Medical Curriculum in Alliance with the Harvard Medical School (Boston/USA)” (1998-2005). He was elected Dean of Education in 1999 and for a second term until 2007. In 2004 he was Adjunct Professor, Burapha University, Thailand and in 2005 Adviser to the Dean, Faculty of Medicine, Naresuan University, Thailand. From 2007-2009 he was Dean of Education for the preclinical part of medical education and representative for international programs of Dresden Medical School. In 2014 he was elected President of the Association of Medical Schools in Europe (AMSE). In October 2017 he retired from his Professorship and was appointed Senior Professor at Carl Gustav Carus Medical School, Dresden, Germany.

It appears nearly impossible to fully describe the many activities in medical education Peter Dieter was engaged in, at home and abroad. And how he managed to develop his reputation as an academic teacher on top of leading successful research projects in all these years. The obituary of his medical school, who most certainly knew him best, has the best insights into these aspects, and will be cited here in part (citation accessed January 27, 2020 at https://tu-dresden.de/med/mf/die-fakultaet/newsuebersicht/ein-leben-fuer-die-wissenschaft):

*“Not only that many students liked his open attitude, he was the type of professor, who willingly allowed people a glance backstage and who exchanged views with everybody. He was a self-confessed fan of the Bee Gees and had a soft spot for “Pfälzer” wine and coffee, spent many hours on his bicycle and in meditation to allow him room for the generation of ideas for numerous top-quality published papers and for his work on boards and in associations. Through his work on national and international boards he contributed to a public discussion of medical education throughout Germany. He significantly participated in the implementation of a multi-faculty curriculum “Master of Medical Education” and promoted the trade mark “Higher Medical Education in Germany” internationally. Until 2008 he was a member of the “Medical Graduate Education” committee of the Professional Medical Association of Saxony and since 2002 a member of the board of directors of the “Academy of Medical Education” of the Association of German Medical Faculties (MFT). His commitment was reinforced by numerous national and international awards. He was honoured by Burapha University, Faculty of Public Health, Thailand und Patan Academy of Health Science, Nepal. In 2010 he was awarded the prize for excellence in medical education by the “Association of German Charitable Foundations” *[end of citation]*.*

This award was, among many other honours and awards, very special to him. The “Ars Legendi” Faculty Prize for Excellence in Teaching is offered jointly by the “Association of German Charitable Foundations” and the Association of German Medical Faculties (MFT), and the award ceremony took place during the 71^st^ Regular Medical Faculties’ Assembly on June 3, 2010 in Halle, Germany. Peter Dieter was the first laureate of this award, together with Jürgen Schäfer from Marburg University. The prize money is 30.000 €, and for Peter Dieter the following laudation was put forward (citation accessed January 27, 2022 under http://www.mft-online.de/presse-standpunkte/telegramm/ars-legendi-fakultaetenpreis-fuer-exzellente-lehre-in-der-medizin-2010): 

*“Prof. Dieter is known for his special innovative and student-centred teaching in biochemistry and always achieved top marks in teaching evaluations. In his medical school Professor Dieter significantly participated in the development of the forward-looking reform-curriculum DIPOL. This curriculum aims to help students to find an easier start into clinical practice through its interdisciplinarity and practical orientation. Also, Professor Dieter facilitated the international exchange for medical clerkships and elective final year clerkships through the establishment of numerous exchange partnerships in Dresden, for example with Harvard Medical School. It is therefore no wonder that the students experience the laureate as a teacher, who grippingly presents the foundations of his discipline, who can be easily approached at any time with problems or projects and receives the respect of his audiences for his outstanding competence. Along with his strong commitment to his faculty proper, Professor Dieter was also one of the first participants in the implementation of the “Master of Medical Education”. He is the most senior member on the steering board of the Academy for Medical Education (AHM). His contributions to these establishments and the co-ordination of a module with a focus on “Leadership and Faculty Development” are important parts of the Master curriculum. His contributions on the board of directors of the German Association for Medical Education (GMA), in the Association for Medical Education in Europe (AMEE) and in further professional societies complete the image of an award-winning scientist, who with great perseverance committed himself to the improvement of the framework conditions of medical education.”* [end of citation].

The way he expressed his thanks for this honour was characteristic for Peter (citation accessed on January 27, 2020, under http://www.mft-online.de/files/seite_142.pdf): 

*“I would like to thank you all cordially for this prize, on behalf of all colleagues who have helped me – who of course also share this prize – and who have helped me with the reforms of the DIPOL^®^ programme in Dresden and have actively supported me in this endeavor. Without these colleagues I would not be standing here today.” *[end of citation]. 

 Personally, he had repeatedly mentioned regarding his work in Dresden, that the Medical Director of Dresden University Hospital Carl Gustav Carus, Prof. Michael Albrecht significantly supported the costly co-operation with Harvard International, the quality management programme for medical education including the (in Germany at that time) unique certification DIN EN ISO 9001:2000 and – together with the medical school – contributed substantial funds.

As Dean for Education he insisted on motivating medical students, sometimes in unconventional ways. For example, by staging singing events with his wife, using scientific texts of his own and set to familiar melodies to inspire students for problem-oriented learning (POL)! 

In spite of his illness he continued to lecture and participated on boards and panels, particularly in his capacity as president of the Association of Medical Schools in Europe (AMSE). In this function he has prepared and participated in the European Annual Congress of the AMSE on November 7-8, 2019 in Lodz, Poland, jointly with the “Medical Board of Medical Assessors”. This probably was – less than 7 weeks before his death –his last mission for the advancement and quality of medical education.

The GMA will dearly miss Peter Dieter and remember him as a professional, as a friend and as a humble colleague. His lifetime achievements have sustainably advanced the quality and importance of medical education in German medical faculties and abroad.

*Eckhart G. Hahn, President GMA 2003-2011*

## Competing interests

The author declares that he has no competing interests.

